# Effects of a Fully Immersive Virtual Reality Exercise Game on Fear of Movement, Pain, and Disability in People with Chronic Non-Specific Low Back Pain: A Pilot Randomized Controlled Trial

**DOI:** 10.3390/healthcare14162641

**Published:** 2026-08-20

**Authors:** Hana Alsobayel, Kholoud Almufaireej, Dalia Alimam, Hamad Alduaij, Afaf Shaheen, Hend Al-Khalifa

**Affiliations:** 1 Department of Health Rehabilitation Sciences, College of Applied Medical Sciences, King Saud University, Riyadh 11433, Saudi Arabia; 2 Department of Physical Therapy, Security Forces Hospital, Riyadh 11481, Saudi Arabia; 3 Basic Science Department, Faculty of Physical Therapy, Cairo University, Cairo 12511, Egypt; 4 Information Technology Department, College of Computer and Information Sciences, King Saud University, Riyadh 11451, Saudi Arabia

**Keywords:** low back pain, virtual reality, video games, fear avoidance, exercise adherence

## Abstract

**Highlights:**

**What are the main findings?**

- A fully immersive virtual reality (VR) exercise game resulted in a statistically significant reduction in pain (NPRS) compared to conventional exercise in individuals with chronic non-specific low back pain (LBP).
- No significant improvements were observed with regard to fear-avoidance beliefs (FABQ) or disability-related outcomes between groups.

**What are the implications of the main findings?**

- VR-based exercise may serve as an effective, engaging modality for short-term pain management in LBP rehabilitation.
- Addressing fear, beliefs, and disability will likely require VR to be integrated with broader biopsychosocial and education-focused rehabilitation approaches.

**Abstract:**

**Background/Objectives:** Low back pain (LBP) is a leading cause of disability worldwide, and exercise-based rehabilitation is recognized as an effective management strategy. However, adherence to exercise remains challenging. Fully immersive virtual reality (VR) exercise interventions may enhance engagement and clinical outcomes. This study aimed to examine the effect of a fully immersive VR exercise game on pain, fear-avoidance beliefs, and disability in individuals with chronic non-specific LBP. **Methods:** A single-blinded randomized controlled trial was conducted, involving 28 participants aged 18–60 years with chronic non-specific LBP. Participants were randomly allocated to either a VR exercise group with standard education (*n* = 14) or a conventional home exercise group with standard education (*n* = 14). Both groups performed their assigned intervention once per day for two weeks. Outcomes included the Fear-Avoidance Beliefs Questionnaire (FABQ), Numeric Pain Rating Scale (NPRS), Oswestry Disability Index (ODI), Back Beliefs Questionnaire (BBQ), and functional performance tests (six-minute walk test, repeated sit-to-stand, and trunk flexion). Measures were assessed at baseline and after a 2-week intervention period. Exercise adherence was recorded using a self-reported log. **Results:** No significant within- or between-group differences were observed for FABQ scores. However, a significant between-group difference was reported for pain reduction in the VR group (*p* = 0.044). No significant differences were reported for disability or belief-related outcomes, or for physical performance outcomes, including the 6 min walk test, repeated sit-to-stand test, and trunk flexion test. **Conclusions:** A fully immersive VR exercise intervention resulted in greater short-term pain reduction compared to conventional exercise in individuals with chronic non-specific LBP. However, it had little impact on fear-avoidance beliefs or disability, highlighting the need for multimodal approaches that address broader biopsychosocial factors.

## 1. Introduction

Low back pain (LBP) is considered among the most common causes of disability in adults worldwide [1]. About 80 percent of the population will experience LBP at least once in their life [2]. Chronic LBP poses a significant public health burden, with an estimated 83 million healthy years of life lost annually due to LBP-related illness and disability [3].

The risk of a high level of disability increases with chronic LBP [4]; for example, in one study, a strong association between chronic LBP intensity and disability was demonstrated in a Saudi population [5]. Some people with LBP may have negative beliefs regarding their pain and tend to avoid physical movement, thus exacerbating their pain and disability [6]. However, among some patients, pain-related fear is a common-sense response that aims to protect a “damaged” back and avoid further LBP [7]. A recent study involving a Saudi population with chronic LBP found that fear avoidance is associated with a risk of high-level disability [8]. Furthermore, a reduction in the fear-avoidance score has been correlated with a reduced disability level [9]. Therefore, treatment strategies that address fear avoidance may be important for reducing the prevalence of chronic LBP-related disability.

According to clinical practice guidelines for managing LBP, physical exercises are one of the most effective rehabilitation methods [10,11]. However, patients with LBP report difficulties adhering to these exercises. This may be due to factors such as the complexity of the exercise program, boredom, lack of supervision and follow-up and fear of worsening their LBP [12,13].

One method that may help overcome the fear of movement and increase adherence to exercises is the use of virtual reality (VR) interventions. Providing an enjoyable and entertaining interactive video gaming-based exercise might help to break the fear cycle by providing a distraction from pain-related thoughts through the immersive virtual environment, thus encouraging the patient to perform the physical activity/exercises [14]. Due to their immersive and interactive nature, VR applications have been shown to be effective at diverting attention away from pain [15,16]. For example, immersive VR has proven effective in reducing pain during medical procedures such as wound care [17], needle-related procedural pain, chemotherapy [18], and dressing changes in patients with a hand injury [18]. VR interventions have also been used to successfully treat chronic pain [19] and improve adherence with home exercises [20]. VR has already been tested directly in patients with chronic low back pain, with mixed—but encouraging—results. A recent meta-analysis pooling 25 randomized trials (over 2600 patients) found that certain VR formats—shooting games and equestrian-style training in particular—led to meaningful reductions in pain and fear of movement compared to standard care, though the picture was less clear when it came to functional disability [21]. This suggests that not all VR interventions work the same way, and different modalities seem to engage patients’ movement and psychology in different ways.

The aim of this study was to investigate the effectiveness of a fully immersive VR exercise game, utilizing a smartphone with motion sensors, in patients with non-specific chronic LBP to address their fear of movement, pain-related disability, physical function, and exercise adherence.

## 2. Materials and Methods

### 2.1. Study Design

A single-blinded, parallel-group pilot randomized controlled trial (RCT) was conducted, with participants randomly allocated to an intervention or control group using a computer-generated permuted block randomization sequence method [22]. A fixed block size of four was used to maintain balanced allocation between the two groups, and the possible allocation sequences within each block were randomly ordered using the RAND function. The allocation sequence was prepared prior to participant recruitment and concealed in sequentially numbered, sealed opaque envelopes. Following the completion of the baseline assessment by the blinded outcome assessor and confirmation of eligibility, the intervention provider opened each envelope in sequence to reveal which group each participant was assigned to. The study employed single blinding, with only the outcome assessor blinded to the group assignment. Participants and intervention providers were not blinded due to the nature of the intervention. No participants requested or received a change in group allocation after randomization.

Ethical approval was obtained from the College of Medicine Human Subjects Institutional Review Board (IRB) at King Saud University (No. E-21-5910) and the Security Forces Hospital Accreditation (No. H-01-R069) and registered with ClinicalTrials.gov (Identifier: NCT05267756).

### 2.2. Participants

Participants were recruited from the Security Forces Hospital, Riyadh, Saudi Arabia, from October 2021 to March 2022. This hospital serves the military and has a physiotherapy department that sees patients from different specialties, including orthopedics. Adult patients (aged 18–60 years) presenting with chronic non-specific LBP (duration > 3 months), who did not report any health condition that would restrict their movement or prevent safe participation and were able to use a smartphone to download and independently operate the VR game were eligible for participation. Smartphone proficiency was among the inclusion criteria because the VR intervention required participants to independently operate a smartphone application (e.g., pairing the headset, launching modules, navigating menus) without in-person supervision during home use [23]. The exclusion criteria were as follows: age of >60 years (due to concerns about balance/movement during the interventions); history of spinal surgery, hip arthroplasty, or spinal deformity (e.g., scoliosis); the presence of red flags (e.g., active cancer, recent unexplained weight loss weight, infection); neurological features accompanying the LBP; vestibular system dysfunction; or pregnancy.

**Figure 1:**
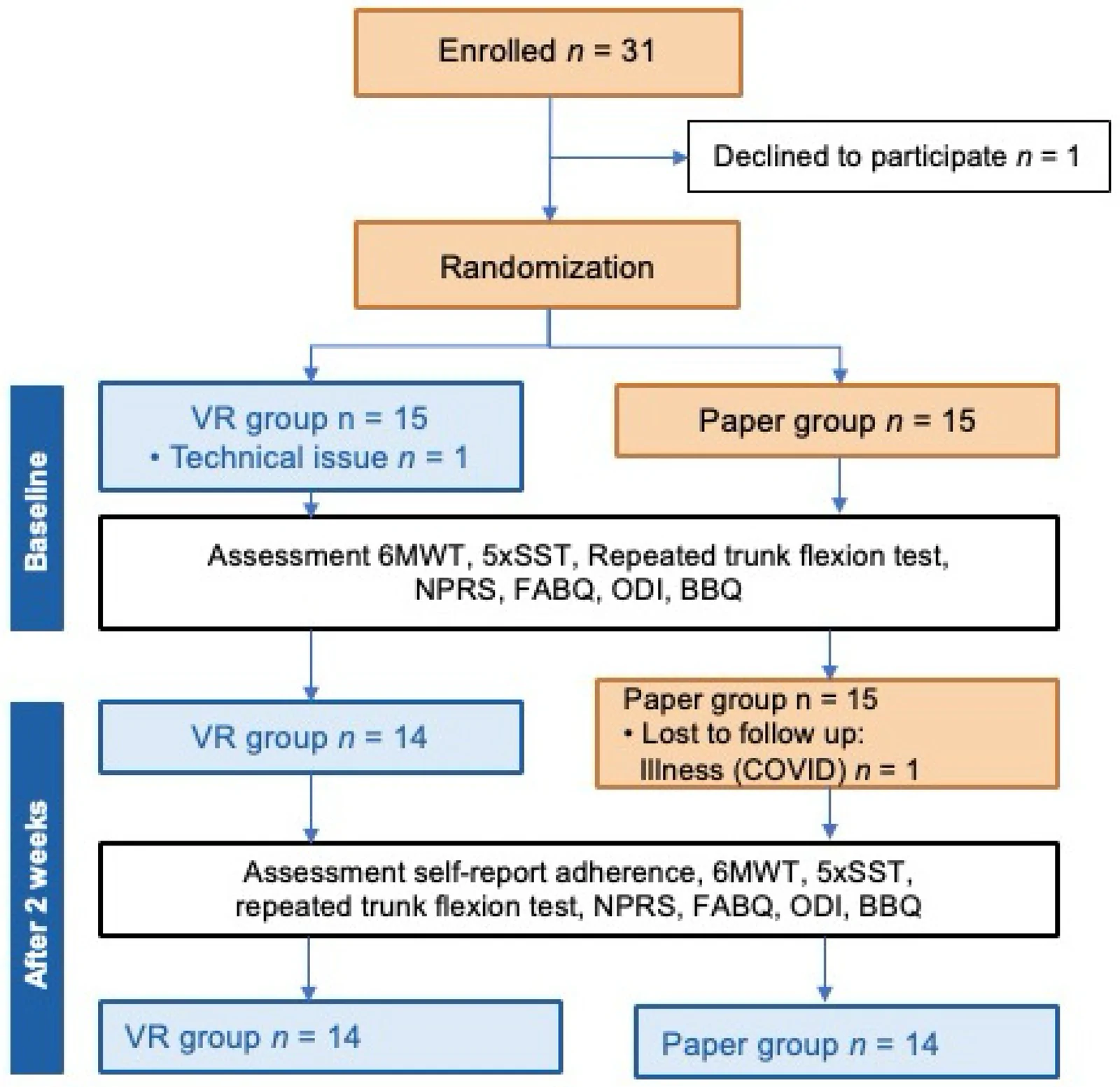
Flow of participants through the study.

PT staff received an email informing them about the aims of the study and the need to recruit patients with chronic non-specific LBP from a walk-in clinic or waiting list. When a patient was referred by an orthopedic clinic or family medicine and came to the physiotherapy department, therapist assistants examined the patients to assess their eligibility. An information sheet was provided to potential participants and an informed written consent form was obtained from those who were willing to participate. The distribution of patients in the control and intervention groups is shown in Figure 1.

### 2.3. Intervention

**Figure 2:**
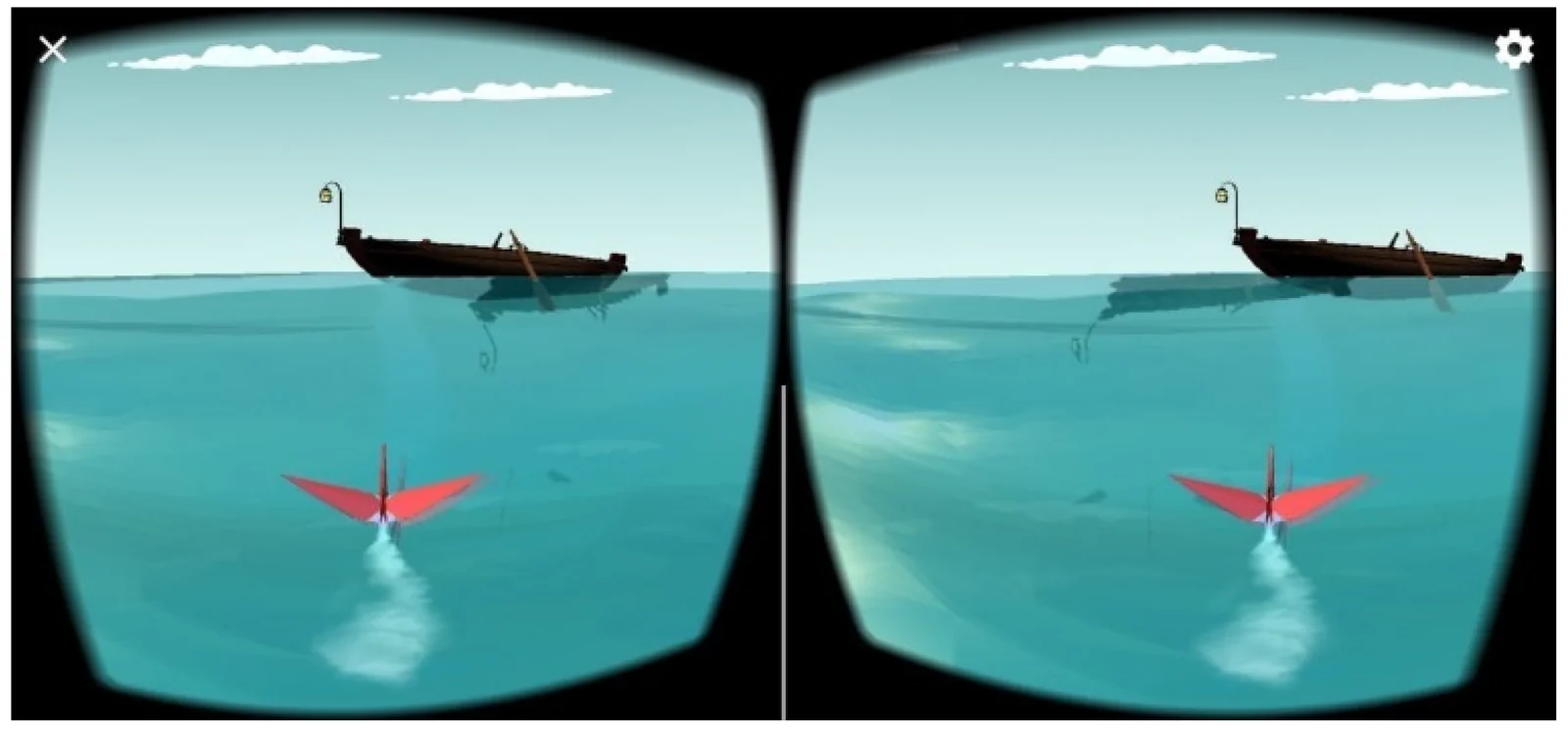
Virtual reality bird game. Note: Kurki was developed by the authors; not commercially available.

Participants in both groups were instructed to perform their interventions once per day for the 2-week intervention period. This duration was chosen based on a previous study which found that a 2-week VR program for chronic musculoskeletal pain produced significant short-term reductions in pain and anxiety [24]. Each exercise session lasted approximately 30–40 min. Before commencing the home-based intervention, all participants attended an initial face-to-face supervised session with the intervention providers, during which the complete treatment plan was explained and participants were trained and supervised to ensure that they were performing the prescribed exercise correctly. Following this initial supervised training session, participants completed their assigned interventions independently at home. All participants were provided with information, advice and behavior modification techniques for LBP based on the latest clinical practice guidelines [10,11]. For example, they were advised on how to reduce worry (e.g., stay active, return to normal activities as soon as possible, avoid worrying, and use strategies to cope with LBP). Participants also received pamphlets produced by the Saudi Spine Society that contained information about LBP and the relevant treatment methods. In addition to the above, the control group was given a brochure detailing conventional therapeutic exercises specific to LBP. The program comprised seven exercises: quadruped lumbar flexion–extension (cat–camel), prone press-up, single knee-to-chest stretching, double knee-to-chest stretching, lower trunk rotation, supine bridging, and standing lumbar extension. Exercises were performed for 10 repetitions, with specific hold times where applicable; the remaining exercises were completed to tolerance, with bridging progressing to a single-leg variation when appropriate. The intervention group, in addition to the usual care, participated in a VR fully immersive exercise game (Figure 2). Intervention group participants were asked to download the program (kurki-application), given VR glasses (3D VR Headset For Smartphone; Mohammed Alshammari Est., Riyadh, Saudi Arabia) and taught how to use the application in a trial session. Participants could perform three LBP therapeutic exercises whilst playing a single game. The game’s settings could be changed to meet each patient’s needs and each participant was able to create a profile to keep track of their exercise history. The game included three difficulty levels; participants were required to successfully complete the required range of motion before proceeding to the next level. The game application was supported on IOS and Android mobile platforms in Arabic and could be played solely using a smartphone with VR cardboard or with VR glasses, which is a low-cost VR intervention. Adherence in both groups was monitored using a daily self-reported exercise logbook throughout the 14-day intervention period.

The VR gameplay consists of various challenges. For example, in order to move the bird in the game (Figure 1), the player has to perform four movements, each consisting of a therapeutic exercise for LBP (Table 1). The game has three difficulty levels, and each level requires the player to perform the movement in a specified range of motion (ROM). Table 2 shows the degree of the angle for each exercise in each level. The angle degrees are selected based on the active ROM.

### 2.4. Outcome Measures

Two physiotherapists, one male and one female, were thoroughly trained and standardized as outcome assessors. This involved a comprehensive review of measurement protocols for all outcome tools, practice sessions, and inter-rater reliability checks to ensure consistent administration and scoring. Demographic information (e.g., gender, age, body mass index) and other outcome data were collected at baseline and after the 2-week intervention period.

The primary outcome was an Arabic version of the Fear-Avoidance Beliefs Questionnaire (FABQ). The secondary outcomes were the Numeric Pain Rating Scale (NPRS), Oswestry Disability Index (ODI), Back Beliefs Questionnaire (BBQ), six-minute walking test (6MWT), repeated sit-to-stand (5xSTS), and repeated trunk flexion, along with the level of adherence to the home exercise program.

The FABQ is a self-reported questionnaire that focuses on how a patient’s fear-avoidance beliefs about physical activity and work may affect and contribute to their LBP and resulting disability [25]. It comprises 16 items where patients rate their level of agreement with each statement on a 7-point Likert-type scale (0 = completely disagree to 7 = completely agree). The maximum score is 96; a higher score represents higher levels of fear-avoidance beliefs. The FABQ has two subscales: the work subscale (FABQw) with 7 items (maximum score of 42) and the physical activity subscale (FABQpa) with 4 items (maximum score of 24) [26]. The Arabic version has good test–retest reliability [27].

Pain intensity was measured using the Arabic NPRS, with participants rating their level of pain on an 11-point scale ranging from 0 to 10 (0 = no pain and 10 = the worst possible pain) [28]. The NPRS has been shown to be reliable for assessing LBP severity [29]. The construct validity showed a positive correlation (r = 0.92, *p* < 0.001) with high agreement between both [29]. The Arabic NPRS is a valid and reliable scale for measuring pain levels with good-to-excellent repeatability (ICC 0.89). The SEM and MDC were 0.71 and 1.96, respectively. Significant correlations with the visual analogue scale scores were reported (*p*  < 0.01) [30].

An Arabic version of the ODI, a self-reported questionnaire, was used to assess the level of function (disability) in activities of daily living in individuals with LBP. It consists of 10 everyday activities (e.g., personal care, walking, and social life) with each activity scored from 0 to 5 (0 = least disability; 5 = greatest disability). The total score is calculated as a percentage of disability, with higher scores indicating higher levels of disability (2). Research has concluded that the ODI is a valid, reliable and responsive clinical tool when used to determine the level of disability [31,32]. The Arabic version showed excellent intra-observer reliability (ICC: 0.99). The construct validity was determined using the Roland–Morris Low Back Pain Disability (r = 0.656), with good correlation [33].

An Arabic version of the BBQ was used to assess participants’ beliefs about the consequences of LBP. It is a self-reported questionnaire with 14 items, 9 of which are used to generate a score and five are used as distractors. Each item is scored on a 5-point Likert scale (1 = completely disagree to 5 = completely agree). The score obtained for each item is reversed, with the total score possible ranging from 9 to 45; a higher score is indicative of more pessimistic beliefs about the consequences of LBP. The first validation of the English version [34] has also been translated into Arabic [35], German [36], and Chinese [37]. Most of these methodological studies have shown good test–retest reliability and validity. The Arabic version of BBQ had strong internal consistency (Cronbach’s alpha α = 0.77) and good test–retest reliability (ICC_(2,1)_ =0.88). Its significant relationship with a disability scale supports its construct validity (r = 0.307; *p* < 0.01) [38].

Physical function and endurance were measured using the 6MWT [39]. Participants were asked to walk on a 30 m stretch of unimpeded walkway for six minutes and the distance covered was recorded [40]. The 6MWT is valid, reliable, and responsive when used with a variety of other conditions such as osteoarthritis [41] and stroke [42]. It has excellent test–retest reliability (ICC = 0.95) [43] and validity with gait speed (r = −0.73) [44].

The repeated STS test was used to assess physical performance [45]. Participants were asked to rise from sitting (in a standard-height chair with a straight back) to standing five times as quickly as possible, and the time taken to do so was measured [46]. The repeated STS test has excellent test–retest reliability (ICC range: 0.988–0.995) [47] and validity with the Time Up and Go test (r = 0.64, *p* < 0.001) [48].

Physical performance was also assessed via the repeated trunk flexion test. Patients were asked to flex to the limit of their range from a neutral standing position, then rise up as fast as possible. This movement was repeated 5 times and the time to complete was measured [49]. Previous studies have shown that the velocity and acceleration of motion are slower in patients with LBP during trunk flexion [50]. Test–retest reliability in LBP (ICC = 0.45) and moderate correlation with repeated sit-to-stand (r = 0.47) [45].

Adherence to the home exercise program (VR group or usual care group) was assessed using a daily self-reported exercise logbook completed throughout the 14-day intervention period. Participants in both groups were instructed to record each day on which they completed the prescribed intervention. The total number of completed exercise days was calculated for each participant, with a possible score ranging from 0 to 14 days; a higher number indicated greater adherence.

### 2.5. Statistical Analyses

A required sample size of 28 (14 per group) was calculated using G*power 3.1. software, based on a *t*-test, a statistical probability level of 0.05, and a power level of 0.80. The calculation was based on a previous study that assessed the effect of VR exercises on LBP and used the ODI to measure disability [51], with an effect size of 1.11 [51].

Data were saved into Microsoft Excel (version 2021, Microsoft Corporation, Redmond, WA, USA), coded, and then entered into the Statistical Package for Social Sciences (version 24.0 for Windows; SPSS Inc., Chicago, IL, USA). All statistical analyses were performed using two-tailed significance tests. A *p*-value less than or equal to 0.05 was considered significant. Data were tested for normal distribution using the Shapiro–Wilk test (Appendix A). First, Descriptive Baseline characteristics (demographic data) were divided into quantitative variables, presented as the mean and standard deviation (e.g., age), and qualitative variables, which are presented as numbers and percentages (e.g., gender, body mass index, education level, work).

Within-group differences at baseline and after 2 weeks were measured using parametric statistics for a normal distribution paired sample *t*-test. A non-parametric Wilcoxon signed-rank test was used for non-normal distributions.

The mean and standard deviation, and the mean difference with a 95% confidence interval (CI) between groups, were calculated using the independent sample *t*-test to compare the means of fear of movement, disability-related pain, back beliefs, physical function test, and adherence rate between the two groups during the 14-day intervention period. For non-normal distribution, the Mann–Whitney test was used.

Some results have a non-normal distribution at baseline and a normal distribution after intervention (where one arm is skewed). Both parametric and non-parametric tests were used and provided the same findings for mean, standard deviation, mean difference, and significance level. See Section 3 for details of the Parametric tests.

Within-group differences at baseline and after 2 weeks were measured using paired sample *t*-tests. The magnitude of the effect was classified as small (0.20 to 0.49), medium (0.50 to 0.79) or large (0.8) according to Cohen’s method [52]. Independent-samples *t*-tests were used to compare the means of the FABQ, NPRS and BBQ, the physical function test, and the adherence rate between the two groups.

## 3. Results

Thirty-one patients were evaluated for inclusion. Of these, 28 were selected (Figure 1). One patient declined to participate, one participant in the VR group experienced technical issues, and another participant in the usual care group was unavailable for follow-up due to illness.

Table 3 summarizes the baseline descriptive data for the 28 participants. The mean age was 42 ± 10 for the control group and 36 ± 6 for the VR group. Fifty percent of the participants were overweight in both groups and more than half were workers (57%). A significant baseline difference was observed for age, with participants in the VR group being younger than those in the control group (*p* = 0.038). Although there was a higher proportion of women in the VR group, the difference in sex distribution was not statistically significant. No significant between-group differences were found for the remaining baseline characteristics.

### Effect of Intervention

As summarized in Table 4 and Table 5, the between-group differences in FABQ scores were not statistically significant for either the work subscale (*p* = 0.593) or the physical activity subscale (*p* = 0.227), and the within-group changes over time were similarly non-significant for both subscales in each group. For NPRS, the between-group difference in pain scores was statistically significant (*p* = 0.044), with the VR group reporting lower scores and a large effect size of 0.80 (Table 4). Within groups, the VR group showed a significant reduction in NPRS scores over time (*p* = 0.000), whereas the control group did not (*p* = 0.111) (Table 5).

For ODI, the between-group difference was not statistically significant (*p* = 0.15) (Table 4). Within groups, the VR group showed a significant reduction in ODI scores over time (*p* = 0.00), while the control group did not (*p* = 0.67) (Table 5).

For the 6MWT, no significant between-group difference was observed (*p* = 0.40) (Table 4). Within groups, the control group showed a significant increase in 6MWT scores over time (*p* = 0.019), whereas the VR group did not (*p* = 0.17) (Table 5).

For the repeated STS test and repeated trunk flexion test, no significant between- or within-group differences were observed (Table 4 and Table 5).

For adherence, the between-group difference was 3.0 days, with the VR group completing more days of the intervention than the control group; this difference approached but did not reach statistical significance (*p* = 0.05), with a large effect size of 0.7 (Table 4). Expressed as a percentage of the 14-day intervention period, adherence was numerically higher in the VR group (70.0% ± 21.4%) than in the control group (48.6% ± 32.1%), corresponding to 9.8 ± 3.0 days versus 6.8 ± 4.5 days, respectively. This between-group difference also approached statistical significance (F = 4.7, df = 26, *p* = 0.05), with a large effect size (0.7), suggesting a potentially meaningful difference in engagement that warrants confirmation in a larger sample.

## 4. Discussion

This study investigated the effect of a fully immersive VR exercise game for people with non-specific chronic LBP with regard to reducing fear, pain intensity, and the related disability, improving physical function and adherence to exercise. Overall, the VR game resulted in significantly lower levels of pain (NPRS) compared to the control group, but no other between-group differences were observed for other outcomes. However, there was a significant reduction in ODI scores for the VR group over time (*p* = 0.008), and this is consistent with a recent meta-analysis that showed a significant reduction in disability in favor of VR compared to other, more common treatments such as physiotherapy.

On the other hand, other studies investigating the effect of VR on kinesiophobia found that it resulted in significantly lower scores in the Tampa Scale of Kinesiophobia compared to usual care [53,54,55]. These studies involved patients with chronic non-specific neck pain [56] and chronic non-specific LBP [57]. The reasons why we did not find significant advantages associated with the use of the VR game (except for NPRS) are unclear, but may reflect the characteristics of our participants, the small sample size, the short-term follow-up and the outcome measures that we used. Importantly, the participants in this study generally had negative back pain beliefs. For instance, participants in the VR group had more negative beliefs (mean [SD]: 25.2 ± 5) compared with the control cohort. These negative beliefs about LBP may have contributed to this finding. The literature suggests that adding a VR component to rehabilitation, whether home- or hospital-based, could be a feasible, safe, and well-tolerated approach that may have positive outcomes in both physical, psychological and cognitive domains [24,58,59].

Low back pain is a multidimensional health condition. Negative pain-related beliefs, e.g., fear that performing a normal daily activity may cause pain, may promote activity avoidance and lead to reduced physical function. A possible explanation for the lack of a significant result for fear could be the treatment that was provided in this study, as it does not contain a cognitive component. Although studies indicate that exercises and physical activity play significant roles in the treatment of chronic LBP, unsuitable pain cognitions, such as fear of movement, have a significant impact on pain intensity and disability in patients with chronic LBP [60]. As such, future studies may consider treatments provided to patients with LBP that target those multifactorial components.

In summary, the results of this study provide some evidence that disability related to LBP and pain intensity can be improved with VR. Using the VR system for rehabilitation may be helpful in providing patients with relevant feedback and motivation [61]. A previous study reported that in patients with chronic LBP, a decrease in physical activity would lead to a reduction in back muscle size and strength, eventually resulting in LBP [62]. Further, it has been suggested that strengthening back muscles could facilitate LBP prevention [63]. As such, the VR system may have encouraged physical activity and different forms of exercise, and may have motivated the participants of this study, leading to the significant findings presented in this research.

To our knowledge, this is the first RCT to examine the effect of using a fully immersive VR exercise game to reduce disability-related pain and fear and improve physical function and adherence to home exercise programs in Saudi people with chronic non-specific LBP. This study had a number of limitations, including the small sample size, which was based on the large effect size from previous studies. As such, this study may not have had sufficient power to produce statistically or clinically meaningful results. However, no adverse effects were reported, and VR proved to have a number of potential benefits for LBP rehabilitation, providing a basis for conducting a larger RCT in the future. Another limitation was the relatively short-term 2-week follow-up. The intervention was intensive, comprising 2 weeks of daily sessions, but this was not enough to impact fear of movement. VR usage in rehabilitation settings has been shown to affect cognitive domains such as fear beliefs. However, the short duration of the intervention may have been insufficient to change cognitive function; thus, a longer duration and multimodal approach may be required going forward [60]. Additionally, participants were recruited from a single setting in a military hospital, which limited the study’s generalizability. Also, as a result of randomization, the VR group was younger and mostly composed of females, which may have impacted the results of the study. Additionally, smartphone proficiency was required for inclusion, which may limit the generalizability of our findings to broader populations with lower digital literacy. Given that digital literacy is an established barrier to engagement with app-based health interventions, patients who are unable to independently operate a smartphone may experience greater difficulty with VR onboarding, reduced adherence, or exclusion from similar interventions altogether, potentially widening existing disparities in access to digital rehabilitation tools. Future work should explore simplified interfaces, caregiver- or clinician-assisted setup procedures, or standalone VR systems that do not depend on smartphone use to determine whether the benefits observed here extend to less technologically proficient populations. Adherence was self-reported and therefore subject to recall and social desirability bias, which may have influenced the near-significant, large-effect-size difference observed between groups. The markedly higher variability in control group adherence (SD 32.1% vs. 21.4% in the VR group) also suggests more heterogeneous engagement with the home exercise program, warranting cautious interpretation. Future trials should incorporate objective adherence tracking, such as automated session logs captured by the VR headset, to verify self-reported compliance. Both groups received standardized education based on current clinical practice guidelines prior to intervention, which may have influenced fear-avoidance beliefs and pain-related behaviors independent of the allocated treatment. This shared component could have introduced a floor effect, potentially attenuating detectable differences between groups. While this approach ensured ethical equivalence, future larger-scale trials should consider isolating or controlling for the educational component to allow more precise estimation of each intervention’s independent effect.

Given the rapid pace of development of VR applications and their potential utility in multiple fields, we recommend conducting further physiotherapy-related VR research with more studies and applications, including more trials on the topic addressed here. We recommend conducting more studies with a greater number of participants, as some differences in outcomes may have been non-significant due to the small sample size in our study. We also recommend a longer follow-up period with more frequent measurements for greater accuracy. Finally, Future work should incorporate sensor-based movement detection to enable more objective, continuous data collection than self-reported diaries currently allow. Recent advances in multisensory human activity recognition demonstrate that combining inertial, ambient, and localization sensor data with deep learning classifiers can reliably distinguish locomotion activities, supporting the development of robust activity recognition systems for tracking human movement within VR environments [64]. Complementing this, multidimensional approaches to motor function assessment—such as decomposition-based analysis of muscle synergy patterns during movement—offer objective, quantifiable metrics of motor coordination that go beyond simple kinematic tracking [65]. Furthermore, surface electromyography (sEMG)-based data has been shown to support machine learning-based diagnosis of underlying muscular conditions, suggesting that integrating sEMG sensing with VR-based exercise monitoring could enhance the precision with which therapeutic exercises are tailored and evaluated [66]. Together, these approaches point toward a multimodal sensor-fusion strategy—combining locomotion tracking, motor synergy analysis, and muscular diagnostics—as a promising direction for more objective and clinically precise VR-based rehabilitation monitoring.

## 5. Conclusions

This pilot study found that a fully immersive VR exercise game did not produce statistically significant between-group improvements in fear of movement or pain-related disability compared to usual care in patients with chronic non-specific low back pain in Saudi Arabia, though it did significantly reduce pain intensity [67]. VR-based exercise also showed a non-significant trend toward improved adherence relative to a home exercise program. Given the modest sample size and short intervention period, these findings should be interpreted as preliminary; larger, adequately powered trials with longer follow-up are needed to confirm whether immersive VR exercise meaningfully improves fear-avoidance and disability outcomes, and to determine its feasibility across broader patient populations, including those with limited digital literacy [67].

## Figures and Tables

**Table 1:** Participants’ movements during the virtual reality game.

Bird Movement	Exercise Description	Exercise
Rolling; jumping	Standing upright, bend the trunk forward into flexion to make the bird jump, then extend the trunk backward beyond neutral into extension to make the bird roll, moving smoothly between the two positions.	Trunk flexion–extension
Move the bird left and right.	Standing upright with arms extended out to the sides, tilt the trunk sideways toward the right shoulder to move the bird right, return to center, then tilt sideways toward the left shoulder to move the bird left.	Lateral tilt
Rotate the bird left and right.	Standing with hands placed on the hips, rotate the trunk to the right to rotate the bird right, return to center, then rotate the trunk to the left to rotate the bird left.	Trunk rotation

**Table 2:** Game level.

Angle	Exercise	Level
30°	Trunk flexion	1
10°	Trunk extension	
10°	Lateral tilt	
10°	Trunk rotation	
45°	Trunk flexion	2
15°	Trunk extension	
15°	Lateral tilt	
20°	Trunk rotation	
60°	Trunk flexion	3
25°	Trunk extension	
25°	Lateral tilt	
30°	Trunk rotation	

**Table 3:** Baseline descriptive characteristics of the study participants (*n* = 28).

Characteristic	Control Group (*n* = 14)	VR Group (*n* = 14)	*p*-Value
Gender, *n* (%)			0.44
Female	7 (50)	10 (71)	
Male	7 (50)	4 (29)	
Age, years, mean ± SD	42 ± 10	36 ± 6	0.038 *
BMI, *n* (%)			1.000
Normal	2 (14)	2 (14)	
Overweight	7 (50)	7 (50)	
Obese	5 (36)	5 (35)	
Education level, *n* (%)			0.555
Non-educated	1 (7)	0 (0)	
Below high school	3 (21)	2 (14)	
High school	4 (29)	7 (50)	
Diploma	1 (7)	0 (0)	
Bachelor	5 (36)	5 (36)	
Employment status, *n *(%)			1.000 *
Working	8 (57)	8 (57)	
Non-working	6 (43)	6 (43)	

* Significant difference (*p* < 0.05).

**Table 4:** Differences in fear and pain related to disability and physical function and adherence rate between groups.

Outcome Measures	Control Group (*n* = 14) Mean ± SD	VR Group (*n* = 14) Mean ± SD	** Mean Difference	*F*-Value	df	* *p*-Value	Effect Size
FABQpa ^a^	15.4 ± 5.1	12.5 ± 6.9	2.8	1.5	26	0.22	0.4
FABQw ^b^	15.3 ± 11.5	17.6 ± 10.7	−2.2	0.29	26	0.59	0.2
NPRS ^c^	4.9 ± 2.4	3 ± 2.3	1.9	4.4	26	0.04	0.8
ODI ^d^	33.2 ± 14.4	25.8 ± 12.5	7.4	0.28	26	0.15	0.5
BBQ ^e^	28.2 ± 6.7	26.5 ± 6	1.7	0.49	26	0.48	0.2
6MWT ^f^	273 ± 56.4	261 ± 37.1	11.4	0.40	26	0.40	0.2
5xSST ^g^	19.7 ± 7	16.7 ± 4.3	2.9	1.8	26	0.18	0.5
Repeated trunk flexion test (s)	39.2 ± 11.8	31.4 ± 9.7	7.7	3.5	26	0.07	0.7
Adherence (days/%)	6.8 ± 4.5 (48.6% ± 32.1%)	9.8 ± 3.0 (70.0% ± 21.4%)	−3.0	4.7	26	0.05	0.7

Fear-avoidance Beliefs Questionnaire (FABQ), Numeric Pain Rating Scale (NPRS), Oswestry Disability Index (ODI), Back Beliefs Questionnaire (BBQ), six-minute walking test (6MWT), repeated sit-to-stand (5xSST). SD: standard deviation; df: degrees of freedom; * significant difference (*p* < 0.05) between groups according to an independent sample *t*-test. ** Defined as control group minus VR group. ^a^ Range from 0 to 24; higher scores > 15 indicate a high level of fear related to physical activity. ^b^ Range from 0 to 42; higher scores > 34 indicate a high level of fear related to work. ^c^ Range from 0 to 10; higher scores indicate worse pain. ^d^ Range from 0% to 20% = minimal; 21 to 40% = moderate; 41% to 60% = severe; 61% to 80% = patient has no mobility; 81% to 100% = patient bedbound. ^e^ Range from 9 to 45; lower scores indicate more pessimistic beliefs about the consequence of LBP. ^f^ ranges from 400 to 700 m; higher scores indicate a better level of physical function. ^g^ ranges from 11.4 to 14.8 s; higher scores indicate a lower level of physical function.

**Table 5:** Differences in fear and pain related to disability and physical function within groups over time.

Outcome Measures	Control Group (*n* = 14) Mean ± SD		** Mean Difference (95% CI)	* *p*-Value	VR Group (*n* = 14) Mean ± SD		** Mean Difference (95% CI)	* *p*-Value
	Pre	Post			Pre	Post		
FABQpa ^a^	17.5 ± 5.2	15.4 ± 5.1	2.1 (−73–5.0)	0.13	16.0 ± 6.2	12.5 ± 6.9	3.42 (0.26–7.1)	0.06
FABQw ^b^	18.9 ± 10.6	15.3 ± 11.5	3.3 (−2.9–10.0)	0.25	22.4 ± 12.5	17.6 ± 10.7	4.78 (−0.8–10.4)	0.09
NPRS ^c^	5.7 ± 2.4	4.9 ± 2.4	0.7 (−2.8–1.7)	0.11	6.0 ± 1.8	3.0 ± 2.3	3.07 (1.6–4.5)	0.00
ODI ^d^	34.4 ± 18.2	33.2 ± 14.4	1.1 (−4.5–6.8)	0.67	32.5 ± 12.0	25.8 ± 12.5	6.71 (2.0–11.3)	0.00
BBQ ^e^	27.3 ± 5.9	28.2 ± 6.7	−0.9 (−7.2–5.3)	0.75	25.2 ± 5	26.5 ± 6.0	−1.28 (−5.2–26)	0.49
6MWT ^f^	251 ± 49.8	273 ± 56.4	−21.4 (−38.7–−4.0)	0.01	244 ± 42	261 ± 37.1	−16.7 (−41.7–8.3)	0.17
5xSST (s) ^g^	19.3 ± 5.4	19.7 ± 7	−0.3 (−2.8–2.2)	0.79	18.3 ± 3.9	16.7 ± 4.3	1.60 (−0.3–3.5)	0.09
Repeated trunk flexion test (s)	34.2 ± 13.3	39.2 ± 11.8	−4.9 (−11.4–1.5)	0.12	32.5 ± 6.2	31.4 ± 9.7	1.03 (−4.1–6.2)	0.67

Fear-Avoidance Beliefs Questionnaire (FABQ), Numeric Pain Rating Scale (NPRS), Oswestry Disability Index (ODI), Back Beliefs Questionnaire (BBQ), six-minute walking test (6MWT), and repeated sit-to-stand (5xSST). SD: standard deviation; df: degrees of freedom; *: significant difference (*p* < 0.05) over time using a paired sample *t*-test. ** Defined as mean for pre-test minus mean for post-test of each group. ^a^ Range from 0 to 42; higher scores > 34 indicate a high level of fear related to work. ^b^ Range from 0 to 24; higher scores > 15 indicate a high level of fear related to physical activity. ^c^ Range from 0 to 10; higher scores indicate worse pain. ^d^ Range from 0% to 20% = minimal, 21 to 40% = moderate, 41% to 60% = severe, 61% to 80% = patient has no mobility, 81% to 100% = patient is bedbound. ^e^ Range from 9 to 45; lower scores indicate more pessimistic beliefs about the consequence of LBP. ^f^ ranges from 400 to 700 m; higher scores indicate a better level of physical function. ^g^ ranges from 11.4 to 14.8 s; higher scores indicate a lower level of physical function.

## Data Availability

The data presented in this study are available on request from the corresponding author due to ethical restrictions.

## Abbreviations

The following abbreviations are used in this manuscript:

VR	Virtual reality
LBP	Low back pain
NPRS	Numeric Pain Rating Scale
FABQ	Fear-avoidance beliefs Questionnaire
ODI	Oswestry Disability Index
BBQ	Back Belief Questionnaire

## Appendix A

**Table A1:** Tests of Normality.

	Kolmogorov-Smirnov ^a^			Shapiro-Wilk		
	Statistic	df	Sig.	Statistic	df	Sig.
Age	0.109	28	0.200 *	0.942	28	0.123
six-minutes walking test 1	0.137	28	0.193	0.950	28	0.201
six-minutes walking test 2	0.117	28	0.200 *	0.943	28	0.136
Trunk flexion test 2	0.152	28	0.098	0.964	28	0.430
Trunk flexion test 1	0.177	28	0.025	0.928	28	0.053
times sit to stand 1	0.104	28	0.200 *	0.954	28	0.248
times sit to stand 2	0.191	28	0.010	0.876	28	0.003
NPRS 1	0.160	28	0.065	0.947	28	0.170
NPRS 2	0.121	28	0.200 *	0.956	28	0.282
FABQw1	0.121	28	0.200 *	0.946	28	0.159
FABQw2	0.125	28	0.200 *	0.963	28	0.417
FABQph1	0.155	28	0.082	0.933	28	0.073
FABQ ph2	0.127	28	0.200 *	0.967	28	0.492
ODI1	0.181	28	0.020	0.877	28	0.003
ODI2	0.124	28	0.200 *	0.945	28	0.152
BBQ1	0.228	28	0.001	0.849	28	0.001
BBQ2	0.107	28	0.200 *	0.975	28	0.732
Adherence	0.169	28	0.039	0.941	28	0.116

* This is a lower bound of the true significance. ^a^. Lilliefors Significance Correction.
